# Assessing the effects of aging on the renal endothelial cell landscape using single-cell RNA sequencing

**DOI:** 10.3389/fgene.2023.1175716

**Published:** 2023-05-05

**Authors:** Mengke Li, Dongliang Wang, Zhong Liu, Yanjing Huang, Qikai Zhang, Caineng Pan, Yuheng Lin, Li Sun, Yingfeng Zheng

**Affiliations:** ^1^State Key Laboratory of Ophthalmology, Zhongshan Ophthalmic Center, Sun Yat-sen University, Guangdong Provincial Key Laboratory of Ophthalmology and Visual Science, Guangzhou, China; ^2^ Research Unit of Ocular Development and Regeneration, Chinese Academy of Medical Sciences, Beijing, China

**Keywords:** aging, single-cell sequencing, renal endothelial cells, glomerular endothelial cells, inflammation

## Abstract

Endothelial cells (ECs) with senescence-associated secretory phenotypes (SASP) have been identified as a key mechanism of aging that contributes to various age-related kidney diseases. In this study, we used single-cell RNA sequencing (scRNA-seq) to create a transcriptome atlas of murine renal ECs and identify transcriptomic changes that occur during aging. We identified seven different subtypes of renal ECs, with glomerular ECs and angiogenic ECs being the most affected by senescence. We confirmed our scRNA-seq findings by using double immunostaining for an EC marker (CD31) and markers of specialized EC phenotypes. Our analysis of the dynamics of capillary lineage development revealed a chronic state of inflammation and compromised glomerular function as prominent aging features. Additionally, we observed an elevated pro-inflammatory and pro-coagulant microenvironment in aged glomerular ECs, which may contribute to age-related glomerulosclerosis and renal fibrosis. Through intercellular communication analysis, we also identified changes in signaling involved in immune regulation that may contribute to a hostile microenvironment for renal homeostasis and function. Overall, our findings provide new insights into the mechanisms of aging in the renal endothelium and may pave the way for the discovery of diagnostic biomarkers and therapeutic interventions against age-related kidney diseases.

## 1 Introduction

As individuals age, there is an acceleration in the dysfunction of endothelial cells (ECs). This deterioration is closely linked to several health conditions, including atherosclerosis, coronary artery diseases, tumor growth, diabetic complications, neurodegenerative diseases, and chronic kidney disease (CKD) (Roumenina et al., 2016; Potente and Makinen, 2017). ECs play a vital role in regulating intercellular communication through their response to secreted cytokines and immune cells within the peripheral blood and renal micro-environments. They are considered a crucial component of blood vessels and act as a protective barrier between blood and underlying tissues.

The kidney is a highly vascularized organ that is composed of diverse populations of endothelial cells. These cells are found in distinct vascular beds, such as arteries, veins, and capillaries, and each population has its own unique transcriptomic signature that is specific to its function (Verma and Molitoris, 2015; Shavlakadze et al., 2019; Kalucka et al., 2020). Renal endothelial cells play an important role in regulating blood flow to local tissue beds, modulating coagulation, inflammation, and vascular permeability (Jourde-Chiche et al., 2019). The functional units of the kidney filtration apparatus are the glomeruli, which are composed of podocytes, endothelial cells, mesangial cells, and a basement membrane (Chiang and Inagi, 2010; Karaiskos et al., 2018).

Glomerular ECs play a crucial role in the glomerular filtration barrier and the preservation of podocyte structure (Satchell and Braet, 2009). These cells are highly fenestrated and have a thick filamentous glycocalyx lining. It has been well established that the homeostasis of renal endothelial cells is closely linked to many forms of CKD, which tend to increase significantly with age, such as diabetic kidney disease and arteriolar nephrosclerosis (Bekassy et al., 2018; Jia et al., 2019; Roumeliotis et al., 2020; Cohen et al., 2021). Despite this strong association between renal endothelial dysfunction and age-related kidney diseases, the specific cellular and molecular changes that occur within each subtype of senescent endothelial cells leading to kidney dysfunction are still not fully understood (Nitta et al., 2013; Roumeliotis et al., 2020; O'Sullivan et al., 2017; Sturmlechner et al., 2017).

The emergence of single-cell RNA sequencing (scRNA-seq) technology has opened up new possibilities for investigating the transcriptomic changes that occur during aging at a single-cell level (Hwang et al., 2018). In this study, we employed scRNA-seq to examine the aging-related changes in the transcriptomic landscape of renal ECs in mice. Our results revealed significant differences in the composition, gene signatures, enriched pathways, transcriptional regulatory networks, differentiation programs, and intercellular communication between young and old mice. This research contributes to our understanding of aging-related changes in renal endothelial cells and provides potential new targets for therapy and diagnostic biomarkers for age-related kidney diseases.

## 2 Materials and methods

### 2.1 Animals and renal endothelial cells collection

C57BL6/J young (2-month-old, 4 animals) and aged (2-year-old, 4 animals) mice were housed in the Animal Management Center of Zhongshan Ophthalmic Center from birth. The experimental procedures were approved by the Institutional Animal Ethics Committee of Zhongshan Ophthalmic Center, Sun Yat-Sen University under Permit ID, 2019–043.

Kidney tissues were obtained from both young and aged mice using the methods described previously (Dumas et al., 2020; Kalucka et al., 2020). Briefly, the process involves perfusing the mice, removing the kidney capsules, and surgically dissecting the kidney tissues under a microscope. The tissues were then chopped into small pieces and digested in a HBSS-based digestion buffer containing 0.2% collagenase I and 100 IU/ml DNAse I. The samples were incubated for 40 min at 37°C, with shaking every 10 min and the digestion was stopped by adding ice-cold HBSS. The digested tissue suspensions were filtered through 70 μm and 40 μm cell strainers and then centrifuged at 300 g for 7 min. The cell suspension was then further enriched for ECs by using mouse CD31 microbeads (Miltenyi Biotec, Cat#130–097–418) following the manufacturer’s instructions. The CD31 enriched single cell suspension was washed with wash buffer (0.5% BSA (Sigma-Aldrich, Cat#10735096001), 2 mM EDTA in PBS (Thermo Fisher Scientific, Cat#14190–094)) and incubated with CD31-FITC (Thermo Fisher Scientific, Cat#11–0311–85), CD45-PE-Cy7 (Thermo Fisher Scientific, Cat#25–0451–82) and viability dye eFluor 450 (Thermo Fisher Scientific, Cat#65–0863–18). Doublets were gated out prior to fluorescence-activated cell sorting (FACS) sorting. Viable CD45-negative and CD31-positive ECs were then sorted into collecting medium containing 10% FBS (Thermo Fisher Scientific, Cat#A38401).

### 2.2 Sequencing and quality control of scRNA-seq data

Single cell suspensions of freshly isolated renal ECs were resuspended in PBS containing 0.04% ultra-pure BSA. scRNA-seq libraries were prepared using the Chromium Single Cell 3’ Reagent Kits v2 (10x Genomics). The cell recovery for each library was 3000. We used Illumina HiSeq4000 to sequence the libraries, and analyzed the mouse genome using CellRanger (10x Genomics, version 3.1.0). The sample data was then aggregated using the CellRanger software, and the resulting expression matrix was further processed using the Seurat R single cell package (version 4.1.0). Cells with fewer than 500 genes (low quality) or more than 5000 genes (possible doublets), or a mitochondrial gene ratio greater than 5% were excluded from the analysis. In the end, a total of 7,381 renal ECs from young mice and 7,432 renal ECs from old mice with high quality were further analyzed. The data was normalized using the NormalizeData function, followed by the Seurat FindVariableFeatures function as implemented in the Seurat package.

### 2.3 Renal ECs clustering and identification

We used the “Harmony” function to integrate transcriptional data from young and old renal ECs into a combined Seurat object. After integrating and scaling the data, we conducted a principal component analysis (PCA) on highly variable genes to identify appropriate principal components for downstream analysis. We then applied the “RunUMAP” function for dimensional reduction, followed by the “FindClusters” function (with a resolution of 0.2) to identify clusters using a graph-based clustering approach. We visualized the resulting data using the “DimPlot” function. To identify marker genes for each cluster, we applied the “FindConservedMarkers” function with a cutoff of adjusted *p*-values < 0.05 and |logFC| > 0.25 (Supplementary Table S1) and visualized the results using the VlnPlot and FeaturePlot functions. Finally, we generated a top 30 marker heatmap for each cluster using the DoHeatmap function.

### 2.4 Differential gene expression analysis

We used the Seurat function “FindMarkers” to detect DEGs in renal ECs between young and old samples. The threshold for adjusted *p*-values was set at < 0.05 and |logFC| > 0.25 in Wilcoxon test (Supplementary Table S2). We obtained the genes associated with aging-related diseases by searching the DisGeNET website (https://www.disgenet.org/home/).

### 2.5 GO term analysis

We conducted GO analysis using the R programming software. The gene IDs were converted using the “org.Mm.eg.db” package (version 4.0.5), and further enriched using the “clusterProfiler” package (version 4.0.5). By setting the threshold for *p*-value cutoff to 0.05 and q-value cutoff to 0.05, we identified significant genetic functions that represent the functions of each cluster and DEGs.

### 2.6 Gene set score analysis

We acquired genes related to SASP, inflammatory response and pro-coagulation by manually curated from previous studies (Hoppe and Dorner, 2012; Ma et al., 2020; Tabula Muris, 2020; Gu et al., 2021; Zhang et al., 2021). A summary of all gene sets used in the study can be found in Supplementary Table S4. We calculated gene set scores by analyzing the transcriptome of each input cell against the assembled gene sets using the Seurat function “AddModuleScore”. To analyze the changes in scores between the young and old group, we employed the ggplot2 R package (version 3.3.5) and conducted a Wilcoxon test.

### 2.7 Transcriptional regulatory analysis

The transcriptional regulatory analysis of marker genes was conducted using the DoRothEA package (https://saezlab.github.io/dorothea/) with a summary TF confidence level from A to C. We utilized the VIPER algorithm in conjunction with DoRothEA to estimate TF activity as previously described (Garcia-Alonso et al., 2019).

### 2.8 Pseudotime trajectory analysis

To further analyze capillary lineage cells, including capillary 1, capillary 2, angiogenic and glomeruli ECs, the Monocle2 R package was used. The marker genes of different clusters were employed as ordering genes with a threshold of an adjusted *p*-value less than 0.05 and a logFC greater than 0.25. The DDRTree dimensionality reduction method was applied to construct the trajectory and was plotted in two-dimensional space. Time differentiation-related genes were obtained with a cutoff of a q value less than 1 × 10^–4^.

### 2.9 Immunofluorescence and immunohistochemistry

Two representative kidney tissues from the young and old group were carefully isolated, and then fixed and embedded them with formalin and paraffin, respectively. The formalin-fixed paraffin-embedded murine kidney sections were subjected to immunofluorescence and immunohistochemistry staining as previously described (Kalucka et al., 2020; Wang et al., 2020; Li et al., 2021).

Briefly, sections were deparaffinized in xylene and rehydrated through gradient ethanol (100%–70%). After rinsing in distilled water, sections were microwaved in 10 mmol/L sodium citrate buffer (pH 6.0) 5 times for 3 min each. Upon cooling down to RT, sections were rinsed three times in PBS, permeabilized with 0.4% Triton X-100 in PBS for 2 h and rinsed again in PBS three times. Sections were then incubated with blocking buffer (10% donkey serum in PBS) at RT for 1 h, followed by incubation with primary antibodies overnight at 4°C and secondary antibodies at RT for 1 h. For immunofluorescence, sections were counterstained with DAPI, while immunohistochemistry sections were counterstained with hematoxylin. Immunofluorescence imaging was performed using a Zeiss LSM 780 confocal microscope at ×1000 and ×600 magnification. Immunohistochemistry images were taken with Carl Zeiss 200 microscope at ×630 magnification.

The primary antibodies used are as follows: rat monoclonal anti-CD31 (Biolegend, 102,401) at 1/500, mouse monoclonal anti-SPARCL1 (Santa Cruz Biotechnology, sc-514275) at 1/200, mouse monoclonal anti-p21 (Servicebio, GB12153) at 1/500 and rabbit monoclonal anti-PAI-1 (Abcam, ab28207) at 1/200. Secondary antibodies used were the following: anti-rat-Alexa Fluor 488 (Thermo Fisher, A-21202) at 1/500 for CD31, anti-mouse-Alexa Fluor 546 (Thermo Fisher, A-21202) at 1/500 for SPARCL1, p21 and anti-rabbit coupled to HRP (GE Healthcare) at 1/300 for PAI-1. DAB staining was used to detect HRP-coupled secondary antibodies.

For colocalization experiments, coimmunostaining of p21/CD31 and SPARCL1/CD31 in mouse sections was performed as indicated above. p21 staining was quantified as the number of p21-positive endothelial cells per glomerulus in 10 microscopic fields at a magnification of ×600. PAI-1 labeling was quantified as the percentage of positive glomeruli over the total number of glomeruli in 10 microscopic fields at ×630 magnification.

### 2.10 SA-β-Gal staining

SA-β-Gal staining was performed using a previously published protocol (Debacq-Chainiaux et al., 2009; Geng et al., 2019; Zhang et al., 2021). In brief, OCT-embedded, snap-frozen, unfixed mouse kidney tissues were cryosectioned at a thickness of 15 μm with a Leica CM3050S cryomicrotome, collected on Superfrost Plus microslides (VWR) and kept at –80°C until use. For SA-β-Gal staining, sections were thawed at RT and processed by using Senescence β-Galactosidase Staining Kit (Beyotime, Cat#C0602) as the manufacturer’s recommendation. Images were taken with Carl Zeiss 200 microscope, and the SA-β-Gal-positive areas were quantified using ImageJ.

### 2.11 Statistical analysis

All experimental data were statistically analyzed using PRISM software (GraphPad 8 Software). Results were presented as mean ± SEM. Comparisons were conducted using the two-tailed Student’s t-test. Asterisks are used as indicators for statistical differences for experimental data; *p* values are presented for bioinformatic analyses. *p*-value < 0.05 was considered statistically significant.

## 3 Results

### 3.1 Construction of scRNA-seq atlas of murine renal ECs

**FIGURE 1:**
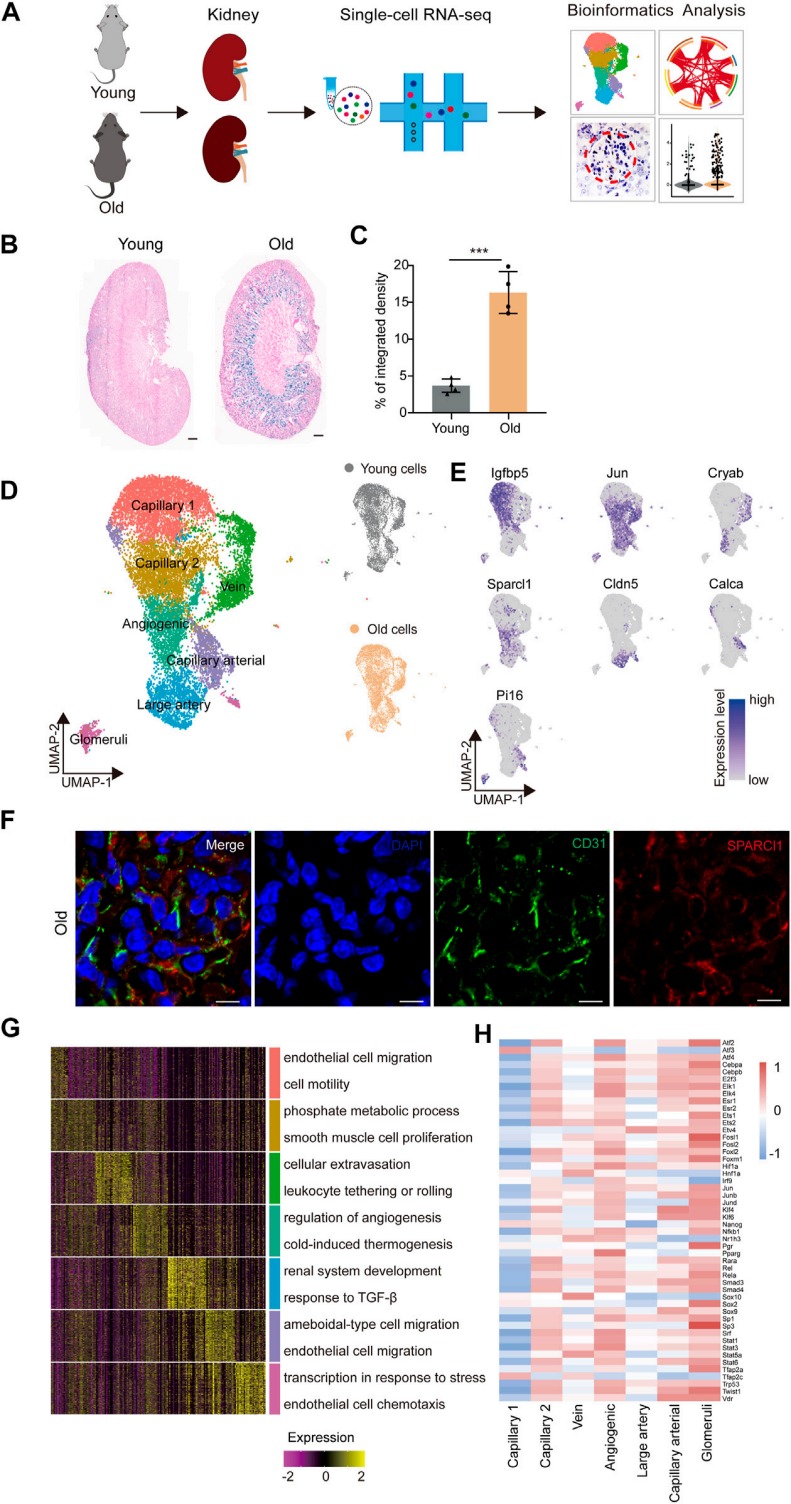
Construction of single-nucleus RNA-seq atlas of the mouse renal endothelial cells (ECs). **(A)** Study flowchart of scRNA-seq and bioinformatics analysis of the mouse renal ECs. Young, n = 4; old, n = 4 mice. **(B)** Representative images of SA-β-Gal staining of the kidney from young and old mice. Scale bar, 500 μm. **(C)** Quantitative data for kidney tissues are shown as means ± SEM. Young, n = 4; old, n = 4 mice. ***p < 0.001 **(D)** Left, UMAP plot showing distribution of different renal endothelial cell types of the mouse. Right, UMAP plots showing distribution of young (top) and old (bottom) mouse renal endothelial cell distribution. **(E)** UMAP plots showing the expression profiles of indicated cell-type-specific marker genes (adjusted *p*-value < 0.05, |logFC| > 0.25) of corresponding cell types in the mouse renal endothelium. **(F)** Representative micrographs of mouse kidney tissue sections, stained for an EC marker (CD31) and Sparcl1 (angiogenic ECs, B), and counterstained with DAPI. Scale bar, 5um. **(G)** Heatmap showing the expression profiles of top 30 cell-type-specific marker genes of different cell types in the mouse kidney with their enriched functional annotations on the right. **(H)** Heatmap showing the cell-type-specific transcription factor activity of mouse renal ECs.

In order to identify age-related transcriptional changes in the renal ECs of mice, we collected kidney tissue samples from young (2 months old) and aged (2 years old) mice, which are roughly equivalent to adult (20 years old) and older human (70 years old) individuals, respectively (Dutta and Sengupta, 2016) (Figure 1A). Additionally, we evaluated the activity of the senescence-associated β-galactosidase (SA-β-Gal) enzyme, which is a widely accepted marker of senescence, and found that the aged group had higher SA-β-Gal activity levels (Figures 1B,C). Renal ECs were further efficiently isolated using fluorescence-activated cell sorting (FACS) and subjected to scRNA-seq using a protocol from 10X Genomics. Four separate batches were analyzed, with each batch consisting of samples from young and old groups (Figure 1A). The goal was to load approximately 3,000 ECs per sample onto the bioinformatic analysis platform. ECs were then selected based on markers specific to ECs (*Cd31*) and unrelated cells, such as smooth muscle cells (*Acta2*), fibroblasts (*Col1a1*), red blood cells (*Hba-a1*, *Hba-a2*, and *Hbb-bs*), pericytes (*Pdgfrb*), and immune cells (*Ptprc*), were excluded. A total of 7,381 young and 7,432 aged single cells were included in downstream analysis after stringent quality filtering and batch correction was applied. The data was then integrated and processed using Harmony (Korsunsky et al., 2019), unsupervised cell clustering methods, and visualized using the Uniform Manifold and Projection (UMAP) algorithm (Figure 1D).

The renal EC populations were found to consist of seven major cell types, as previously reported (Kalucka et al., 2020). These include capillary 1 endothelial cells (*Igfbp5* ^*+*^), capillary 2 endothelial cells (*Ier3* ^*+*^), venous endothelial cells (*Cryab* ^*+*^), angiogenic endothelial cells (*Sparcl1* ^*+*^), large artery endothelial cells (*Cldn5* ^*+*^), arterial endothelial cells (*Calca* ^*+*^), and glomeruli endothelial cells (*Pi16* ^*+*^) (Figure 1E). To verify the scRNA-seq results, we performed double immunostaining using the marker gene *Sparcl1*, and successfully validated the presence of renal angiogenic ECs in the old group (Figure 1F). Furthermore, we investigated whether aged renal ECs exhibit elevated senescence-associated secretory phenotype (SASP) scores, as this is a common feature of senescent cells. Our data show that the aged sample have elevated scores for SASP (Supplementary Figure S1A), and various old renal ECs presented much higher gene set scores when compared to their young counterparts (Supplementary Figure S1B).

The Gene Ontology (GO) analysis of cell-type-specific top 30 marker genes revealed the functional characteristics of the corresponding EC clusters in the murine kidney (Figure 1G). For example, the GO term “epithelial cell migration and motility” was enriched for the top 30 marker genes for capillary1 ECs, “phosphate metabolic process” and “smooth muscle cell proliferation” for capillary2 ECs, and “cellular extravasation” and “leukocyte tethering or rolling” for vein ECs. The GO terms “regulation of angiogenesis” and “cold-induced thermogenesis” correspond to molecular features of angiogenic ECs, indicating their role in angiogenesis (Zhang et al., 2011; Ye et al., 2017). Additionally, the GO terms “renal system development” and “response to TGF-β” were specific to large artery ECs, and “ameboidal-type cell and endothelial cell migration” were enriched for arterial capillary ECs. Glomerular ECs were found to increase the expression levels of genes involved in “transcription in response to stress”, as evident by the marker gene *Pi16*, a shear stress marker (Hazell et al., 2016), and “endothelial cell chemotaxis”, suggesting their essential roles in inflammatory reactions.

We next constructed transcriptional regulatory networks to reveal cell-type-specific TF networks using the software DoRothEA (Garcia-Alonso et al., 2019) (Figure 1H). For example, the transcription factor ATF3, which binds the cAMP response element (CRE) and is involved in DNA damage repair processes (Basu and Krishnamurthy, 2010), was particularly increased in capillary 1 ECs (Allen-Jennings et al., 2002). Additionally, members of the ETS family (ETS1 and ETS2) were shared by both angiogenic and glomerular ECs, and have been shown to regulate vascular development during endothelial cell differentiation, in accordance with previous research (Casie Chetty and Sumanas, 2020; Quijada et al., 2021). Furthermore, the Signal Transducer and Activator of Transcription (STAT) family, which plays a vital role in regulating angiogenesis and cell differentiation (Verhoeven et al., 2020; Chen et al., 2021), also showed increased activity in both angiogenic and glomerular ECs. The majority of transcription factors listed were upregulated in angiogenic and glomerular ECs, indicating that these cells may display multiple functions that are regulated by a complex array of factors. Overall, our study provides a transcriptomic atlas of murine renal ECs at single-cell resolution and offers a comprehensive understanding of the molecular changes induced by aging.

### 3.2 Aging-related cellular and molecular characteristics of murine renal ECs

**FIGURE 2:**
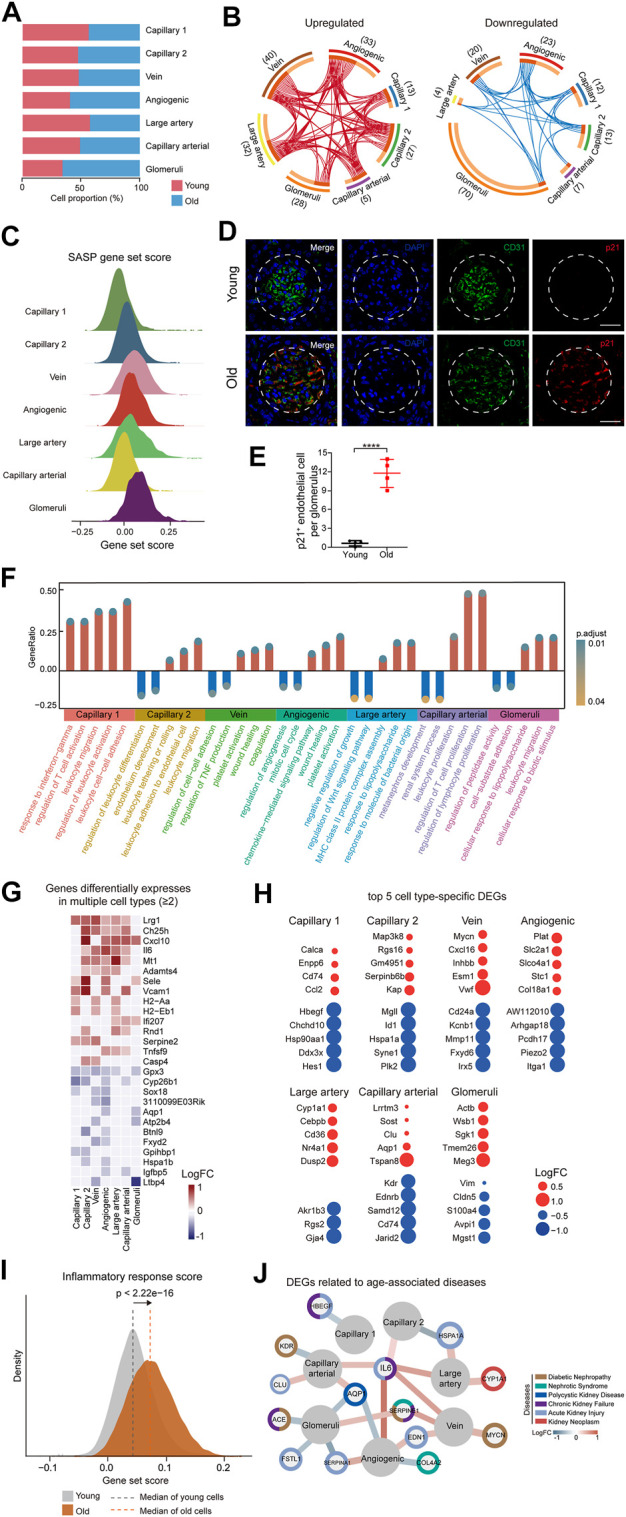
Cellular and molecular aging characteristics of the aged mouse renal ECs. **(A)** Bar plot showing the proportions of different renal ECs from young and old groups. **(B)** Circos plots showing aging-related up and down-regulated differentially expressed genes (DEGs) (adjusted *p*-value < 0.05, |logFC| > 0.25) of different endothelial cell types in the mouse kidney. Each connecting curve represents a gene that is up- or down-regulated in 2 cell types. **(C)** Density plots showing SASP gene set scores of different renal EC types in aged sample. **(D)** p21 and CD31 coimmunostaining in kidneys from young and aged mice. Original magnification ×600. Scale bar, 50 um. Panels are representative images of 4 young and old mice. **(E)** Quantification of p21-positive endothelial cells per glomeruli from young and aged mice. **(F)** Bar plot showing GO terms enriched for aging-related DEGs of different endothelial cell types in the mouse kidney. Y axis represents the ratio of differentially expressed genes to total DEGs in corresponding terms. **(G)** Heatmap showing genes differentially expressed in at least 2 cell types in mouse renal ECs. Only genes with same direction of differential expression among different cell types are included. **(H)** Dot plots showing top five cell-type-specific DEGs of different endothelial cell types. Only those with annotations are showed. Red dots represent upregulated genes and blue dots represent downregulated ones. **(I)** Density plots showing gene set scores of inflammatory response genes in renal endothelial cell types across young and old group. **(J)** Network plot showing DEGs associated with kidney diseases in different renal endothelial cell types.

To investigate the age-related changes in gene expression specific to each cell type in the renal ECs, we initially compared the gene expression patterns of marker genes for each cell type in the renal ECs. Our findings indicated that the expression signatures were similar, suggesting that cell identities remain relatively consistent during the aging process (Supplementary Figure S1C). We then evaluated the proportion of different ECs between the young and old groups. Our analysis revealed that the population of glomerular ECs and angiogenic ECs specifically increased in the old renal endothelium at advanced age (Figure 2A and Supplementary Figure S1D).

In order to identify the cell types most affected by aging, we analyzed differentially expressed genes (DEGs) across 7 cell types. The results showed that glomeruli ECs had the largest numbers of DEGs, with 28 upregulated genes and 70 downregulated genes in aged samples (Figure 2B). We next analyzed the SASP score between different types of old renal ECs and found that glomerular ECs had the highest SASP score (Figure 2C). Furthermore, we conducted co-immunostaining studies using markers for senescence (p21) and endothelial cells (CD31) and confirmed an increase in glomerular ECs senescence during aging (Figures 2D,E).

In addition, we used gene ontology enrichment analysis to identify molecular pathways and biological processes affected by aging across the seven subpopulations (Figure 2F). We found that “leukocyte activation” and “leukocyte cell-cell adhesion” were enriched in capillary 1, suggesting an imbalanced immune function in aging kidneys. For angiogenic ECs, increased “platelet activation” and “wound healing”, pointed to functions of repairing acute and chronic kidney lesions (Landray et al., 2004; Jansen et al., 2018). Additionally, increased expression of genes related to “response to lipopolysaccharide” and “leukocyte migration” in glomerular ECs implied for elevated inflammation with age.

A total of 15 genes were consistently upregulated, and 12 genes were consistently downregulated in at least 2 cell types (Figure 2G). For example, *Cxcl10*, which is upregulated in aged large artery, capillary, and angiogenic ECs, has been linked to progressive renal fibrosis and the recruitment of macrophages into the kidney (Petrovic-Djergovic et al., 2015; Zhang et al., 2018). *Gpx3* was downregulated in various types of ECs in the aged kidney, including large artery, vein, capillary, angiogenic ECs, and glomerular ECs, and may be linked to impaired functions of absorption and secretion in the aged kidney (Burk et al., 2011; Chang et al., 2020).

In addition to identifying DEGs across all subpopulations, we also focused on cell-type-specific DEGs. We identified the top 5 DEGs for each subpopulation and examined their potential connections to cellular dysfunction and tissue pathology (Figure 2H). For instance, *Cd74* and *Ccl2*, the two most highly upregulated genes in aged capillary 1, are involved in renal inflammation and macrophage chemotaxis, which can worsen renal injury, inflammation, and fibrosis (Sanchez-Nino et al., 2013; Cao et al., 2015; Lim et al., 2016). *Sgk1*, which plays a role in recruiting monocyte, was upregulated in glomerular ECs, leading to high levels of vascular inflammation and the formation of atherosclerotic lesions (Yang et al., 2012; Borst et al., 2015).

As analysis of DEGs implied an age-related activation of inflammation, we calculated scores for inflammation response. Our data showed that old group presented much higher gene set scores for inflammation response (Figure 2I).

We next conducted a comparative analysis of age-related DEGs by identifying genes associated with various renal diseases such as diabetic nephropathy, nephrotic syndrome, chronic kidney failure, polycystic kidney disease, and kidney neoplasm from the DisGeNET database (https://www.disgenet.org/home/) (Figure 2J). Our analysis revealed that most of the high-risk DEGs were enriched in glomerular and angiogenic ECs, suggesting that these 2 cell types are more susceptible to acute kidney failure, polycystic kidney disease, and nephrotic syndrome, as demonstrated by the expression of genes such as *Fstl1*, *Edn1*, and *Aqp1*. Additionally, some high-risk DEGs were involved in multiple kidney disorders, such as *Serpine1*. Among these high-risk DEGs, *Il6* and *Aqp1* were dysregulated in more than 2 cell types and may represent potential targets for preventing renal functional decline and chronic kidney diseases in the elderly population.

Overall, our research uncovered aging-related molecular characteristics of renal endothelial cells, demonstrating glomerular ECs and elevated inflammation as the most affected by aging.

### 3.3 Identification of pro-inflammation and pro-coagulation activation in aged glomerular ECs

The glomerulus is a complex bundle of capillaries lined by delicate endothelial cells known as fenestrated endothelial cells. These endothelial cells play a crucial role in filtering circulating blood and creating urine for excretion, which is vital for proper kidney function (Pollak et al., 2014). However, these cells are particularly susceptible to diseases, such as diabetes and hypertension. Additionally, age-related kidney diseases are characterized by the development of glomerular lesions, such as glomerulosclerosis and podocyte loss (Fogo, 2007; Wiggins, 2012; Reidy et al., 2014; Hodgin et al., 2015).

**FIGURE 3:**
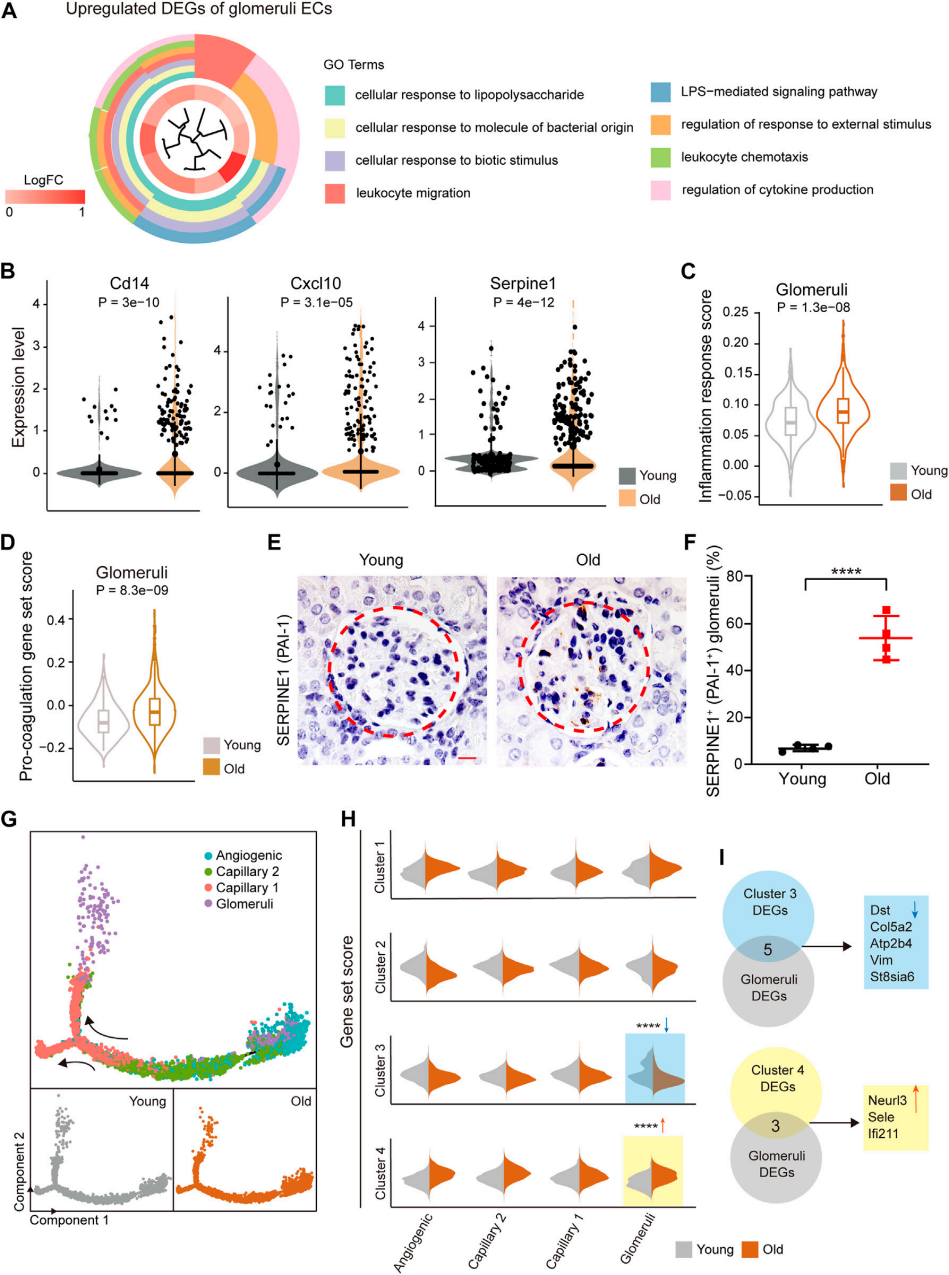
Elevated inflammation and pro-coagulation with age in the mouse glomerular ECs. **(A)** Circle plot showing GO terms of aging-related upregulated DEGs in glomerular ECs of the mouse kidney. **(B)** Violin plots showing expression levels of indicated genes in glomerular ECs of the mouse kidney from young and old. **(C)** Violin plot of inflammatory response score in glomerular ECs of the mouse kidney from young and old groups. **(D)** Violin plot of pro-coagulation gene set score in glomerular ECs of the mouse kidney from young and old groups. **(E)** PAI-1 immunohistochemistry staining in kidneys from young and aged mice. Original magnification×630. Scale bar, 20 um. n = 4 for young and old mice. **(F)** Quantification of PAI-1-positive glomeruli in kidneys from young and aged mice. **(G)** Pseudo-time analysis of the capillary lineage cells in the mouse renal ECs. The points are colored by cell types (top) and age (bottom). The arrows indicate the directions of differentiation trajectories. **(H)** Violin plots showing gene set scores of indicated clusters in different stages of capillary lineage cells of young and old groups. ****p < 0.001. **(I)** Up, pie plot showing overlapped genes between cluster 3 DEGs and aging-related downregulated DEGs of mouse glomerular ECs. Down, pie plot showing overlapped genes between cluster 4 DEGs and aging-related upregulated DEGs of mouse glomerular ECs.

To investigate the transcriptional changes that occur in glomerular ECs with aging, we performed GO analysis of age-related DEGs in the glomerular ECs. We found that the up-regulated genes were enriched for “cellular response to external stimulus,” “leukocyte migration,” and “regulation of chemokine production” in the aged ECs, indicating an increased inflammatory response with age (Figure 3A). Additionally, a group of genes related to inflammation activation, such as *Cd14*, *Cxcl1*, *Cxcl10*, and *Sele*, were found to be up-regulated in the aged glomerular ECs compared to their younger counterparts (Figure 3B and Supplementary Figure S2B). Accordingly, we discovered an age-associated rise of inflammation response scores in glomerular ECs (Figure 3C).

We also discovered that the gene *Serpine1* (serine protease inhibitor) is predominantly expressed in aged glomerular ECs, which aligns with previous research (Cohen et al., 2021) (Figure 3B). SERPINE1, also known as plasminogen activator inhibitor-1 (PAI-1), plays a role in impaired fibrinolysis and the formation of microthrombi. Additionally, glomerular ECs presented elevated pro-coagulation gene set scores with age (Figure 3D). Immunohistochemical experiments showed an increased expression of SERPINE1/PAI-1 in aged glomerulus regions (Figures 3E,F).

In order to investigate age-related molecular disruptions along the differentiation trajectory, we separately ordered the capillary lineage ECs (including capillary1 and capillary2), angiogenic ECs, and glomeruli ECs from young and old groups in a pseudo-temporal manner. We began by studying angiogenic ECs and progressed through capillary 2 and capillary 1 before finally reaching glomerular ECs (Figure 3G and Supplementary Figure S2C). By clustering stage-specific gene expression along the pseudo-time, we identified four distinct clusters of expression profiles (Supplementary Figure S2D and Supplementary Table S3). We next compared the expression of genes specific to different clusters in young and old glomeruli ECs. We found that genes specific to cluster 4 were significantly upregulated and genes specific to cluster 3 were downregulated in aged glomeruli ECs (Figure 3H). By analyzing both cluster-specific DEGs and DEGs related to aging, we identified five downregulated and three upregulated genes shared between aged glomeruli-specific DEGs and cluster specific DEGs (Figure 3I).

In summary, these results suggest that there is a correlation between aging and the activation of inflammation and coagulation, which in turn impairs glomerular function and contributes to age-related diseases.

## 4 Discussion

In this study, we established the first comprehensive single-cell transcriptomic roadmap of aging in renal ECs in mice. By identifying unique transcriptional signatures, we were able to capture a wide range of cell types present in the kidney, including capillary 1, capillary 2, vein, angiogenic, large artery, arterial capillary, and glomerular ECs. This is in line with a previous study by Kalucka et al., who conducted a single-EC transcriptome study using 11 young mouse tissues (Kalucka et al., 2020). Additionally, we used co-immunostaining techniques to confirm the presence of angiogenic ECs in older samples.

We noticed increased senescent cells by SA-β-Gal staining and higher SASP gene set scores in the old mice kidney. Especially, for glomerular ECs, with the highest SASP score, we found the largest number of DEGs, including those annotated as high-risk genes for renal diseases. Our findings may reflect that glomerular ECs were the most susceptible to aging. Gene ontology analysis of DEGs also revealed an increase in inflammation with age, consistent with the idea that aging often leads to a low-grade inflammatory state (Krabbe et al., 2004; Vasto et al., 2007; Donato et al., 2008).

In addition, we observed an upregulation of the inflammatory response score in aged renal ECs compared to young ECs. Inflammation has been well-documented to play a key role in the pathophysiology of CKD and end-stage renal disease (Akchurin and Kaskel, 2015). A variety of factors contribute to this chronic inflammatory status in CKD, including increased production of pro-inflammatory cytokines, oxidative stress and chronic infections. For example, we identified upregulation of many pro-inflammatory cytokines such as CD14, CXCL10, CXCL1, and SELE. CD14 acts as a receptor for bacterial lipopolysaccharide (Gangloff et al., 2005) and plays a role in mediating the innate immune response to bacterial infection (Haziot et al., 1996; Jiang et al., 2005). CXCL10 is a pro-inflammatory cytokine and is known to be involved in a wide variety of processes, such as chemotaxis and activation of immune cells (Gao et al., 2017), making it an important player in viral infections by stimulating the activation and migration of immune cells to infected sites (Lindell et al., 2008; Wuest and Carr, 2008; Srivastava et al., 2017). These cytokines can increase the permeability of endothelial cells and impair the function of the endothelial cell barrier (Lindell et al., 2008; Wuest and Carr, 2008; Gao et al., 2017; Srivastava et al., 2017; Jourde-Chiche et al., 2019).

Our findings also indicate that the glomerular ECs of older individuals have pro-coagulant properties, which is evidenced by the significant increase in the expression of *Serpine1*. Previous research has shown that inflammation and coagulation have a strong interconnection, which is mediated by protease-activated receptors (PARs) in the renal endothelial response to external stimuli (Rezaie, 2014). The activation of SERPINE1(PAR1) by coagulation proteases increases the renal endothelial expression of inflammatory cytokines (such as IL-1, IL-6, and TNF) and cell adhesion molecules (such as E-selectin and P-selectin), which leads to EC apoptosis and compromised renal EC barrier function (Rezaie, 2014). Furthermore, previous studies have confirmed that enhanced PAI-1 expression raises the possibility of glomerulosclerosis and contributes to the fibro-genic process that leads to CKD (Eddy and Fogo, 2006). PAI-1 inhibition has been shown to attenuate experimental kidney fibrosis (Eddy, 2002). A pioneering study demonstrated that senescence drives age-related kidney disease by activating PAI-1 in glomerular ECs, and blocking PAI-1 in senescent endothelial cells protects glomeruli from glomerular lesion development and podocyte loss in aged mice (Cohen et al., 2021).

Through pseudo-time analysis we revealed that the expression profiles of cluster-specific genes between young and old group were mainly enriched in glomerular ECs. *Sele*, which encodes E-selectin and promotes modulation of immune adhesion (Becker-Andre et al., 1992), was upregulated in aged glomerular ECs. Previous research has shown that renal microvascular endothelial cells produce adhesion molecules, such as E-selectin, to promote the attachment of immune cells in the early stage of kidney injury (Nangaku, 2006; Singh et al., 2008). When these glomerular endothelial cells have increased levels of adhesion molecules and are exposed to inflammatory signaling molecules, it results in increased leakage of fluids and proteins through the blood vessel walls and decreased function of the barrier (Jourde-Chiche et al., 2019). Additionally, five genes in cluster 3-specific DEGs were downregulated during aging (*Dst*, *Col5a2*, *Atp2b4*, etc.). These genes are related to intracellular calcium levels, cytoskeleton organization, and extracellular matrix organization (Yang et al., 1996; Lessard et al., 2017), suggesting that there may be glomerular dysfunction as a result of aging.

Therefore, these results indicate an age-related activation of inflammation and coagulation and consequent promotion of a pro-inflammatory and pro-fibrotic microenvironment impairing glomerular function. Therapeutic schemes aimed at reversing the pro-inflammatory and procoagulant phenotype of endothelial cells and slowing glomerular fibrosis may hold promise for the treatment of renal disease.

Our research has a few limitations that should be noted. Firstly, it is acknowledged that the assigned renal EC phenotypes are only based on transcriptomic data. A more comprehensive understanding of the role of each renal EC phenotype would be obtained by a combination of protein analysis and functional experiments. Secondly, to confirm the putative impact of specific glomerular DEGs with aging, it would be beneficial to conduct biological experiments and functional validation. Thirdly, the scope of this study is limited to renal endothelial cells in mice, providing an opportunity for future research to create an all-encompassing atlas of kidney cells in both mice and primates. Finally, it is possible that some endothelial cell types were not detected due to loss during tissue isolation.

In summary, we constructed the first single-cell transcriptomic atlas of murine renal endothelium aging. This provides a comprehensive resource that illustrates molecular signatures at the single-cell level and expands our understanding of related pathologic conditions. Through this atlas, we identified molecular features that correspond to elevated inflammation, coagulation and compromised glomerular function in old kidneys. These features collectively contribute to renal functional decline, providing new insights into the pathological mechanisms and potential therapeutic targets for age-associated kidney diseases.

## Data Availability

The original contributions presented in the study are publicly available. This data can be found here: https://ngdc.cncb.ac.cn/. Accession number: PRJCA014447.
