# Supplementary figures and images for: Assessing the effects of aging on the renal endothelial cell landscape using single-cell RNA sequencing
Source: Front Genet. 2023 May 5;14:1175716. doi: 10.3389/fgene.2023.1175716 (PMC10196692; doi:10.3389/fgene.2023.1175716)

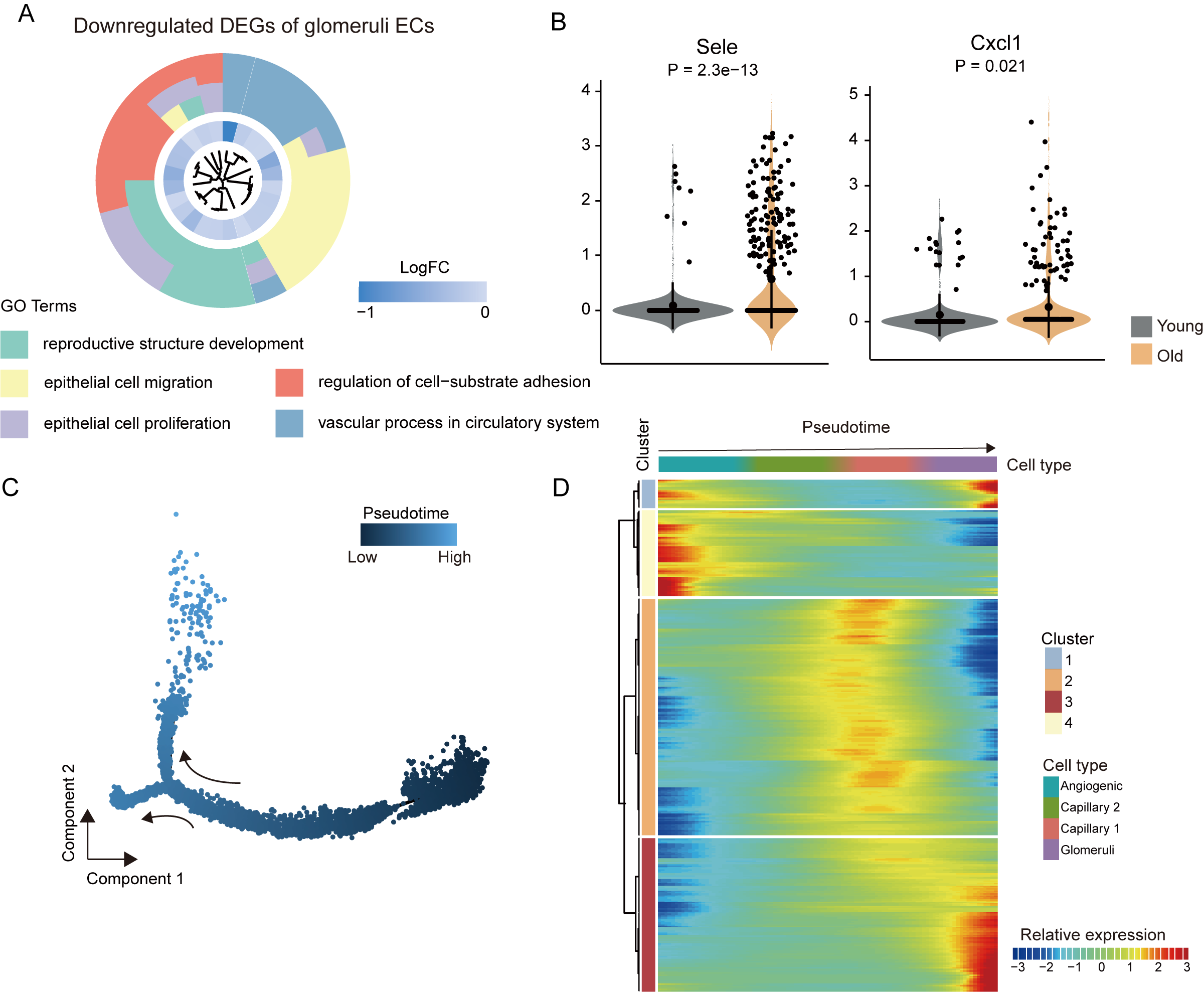

Supplement: Supplementary file 3 [file Image2.TIF]

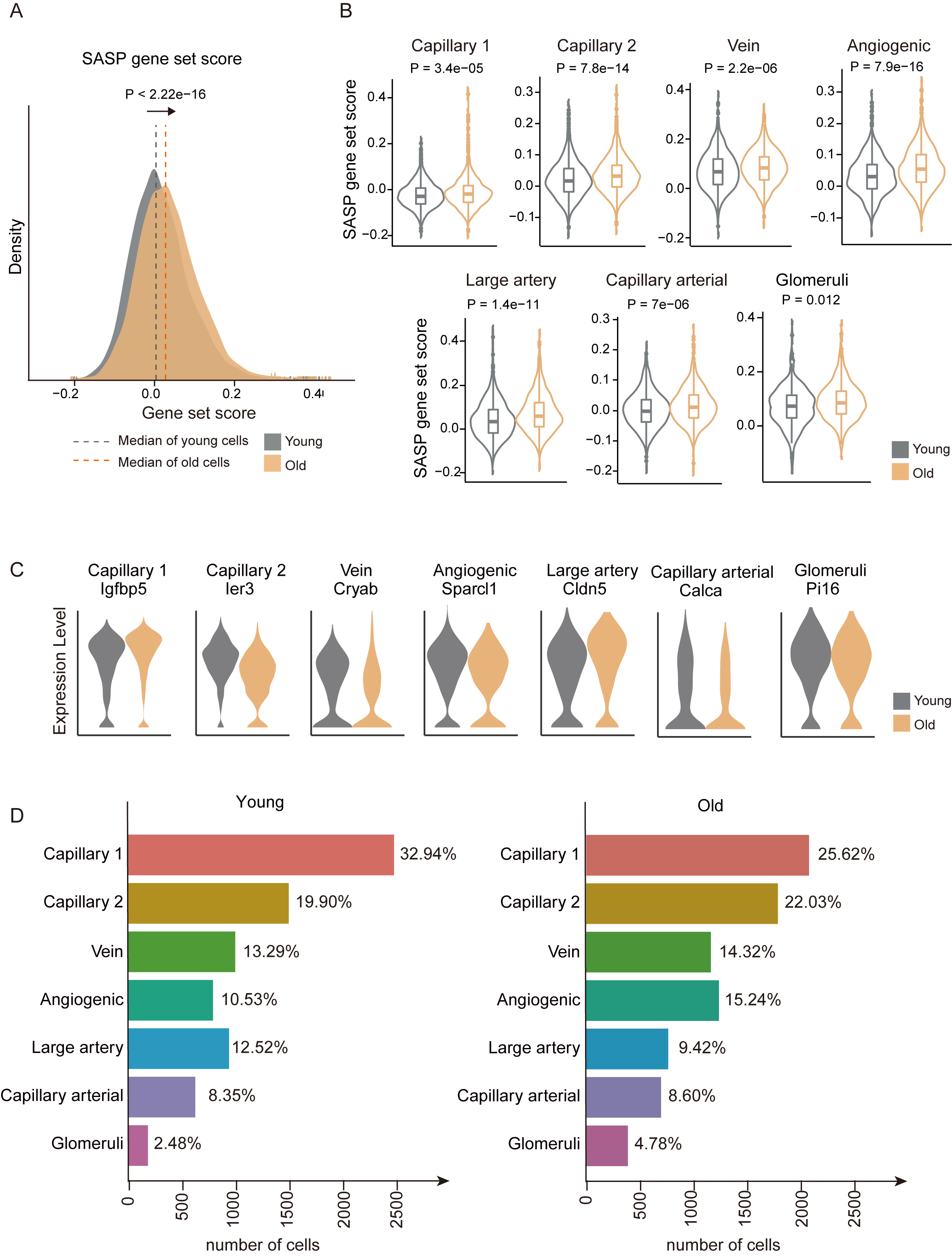

Supplement: Supplementary file 4 [file Image1.TIF]
